# Comparing Rabies Antibody Titres in Imported Dogs to a Population of Dogs in Ontario, Canada

**DOI:** 10.1111/zph.13225

**Published:** 2025-05-14

**Authors:** Catherine R. Belanger, Maureen E. C. Anderson, J. Scott Weese, Kelsey L. Spence, Katie M. Clow

**Affiliations:** ^1^ Department of Population Medicine, Ontario Veterinary College University of Guelph Guelph Ontario Canada; ^2^ Ontario Ministry of Agriculture, Food and Agribusiness Guelph Ontario Canada; ^3^ Department of Pathobiology, Ontario Veterinary College University of Guelph Guelph Ontario Canada

**Keywords:** dog, importation, rabies, serology, vaccination

## Abstract

**Introduction:**

Vaccinating dogs against rabies virus is essential for protecting animal and public health. Most dogs imported into Canada must have a valid rabies vaccination certificate but do not require serological testing to confirm response to vaccination. The objective of this study was to determine the proportion of dogs with rabies antibody titres below 0.5 IU/mL in a sample of imported dogs with rabies certificates, compared to rabies antibody titres in nonimported dogs in Ontario, Canada.

**Methods:**

Serum was collected from a convenience sample of dogs imported via rescues to Ontario, within a month of arrival and before revaccination with rabies in Canada. Rabies antibody titres were measured using the rapid fluorescent foci inhibition test (RFFIT) at Kansas State University's Rabies Laboratory. Univariable logistic analysis examined demographic, vaccine and country of origin factors associated with achieving a titre of at least 0.5 IU/mL. Rabies antibody titres from nonimported dogs were obtained from the University of Guelph's Animal Health Laboratory records. Dogs tested between 2013 and 2023, using either the RFFIT or fluorescent antibody virus neutralisation (FAVN) test, were included.

**Results:**

From October 2021 to November 2022, serum was collected from 67 dogs arriving mainly from Egypt (*n* = 46). In total, 48% (32/67) of these dogs had titres below 0.5 IU/mL, and this included 19 dogs that had no measurable titre. No examined factors were significantly associated with the outcome. Of the 65 nonimported dogs, 14% (9/65) had titres below 0.5 IU/mL.

**Conclusions:**

Rabies titres of many imported dogs were below the international standard for transboundary movement. A high proportion of dogs with unmeasurable antibodies suggests some had not been effectively vaccinated. Veterinarians should be aware of this gap and consider revaccinating imported dogs to protect both animal and public health, and to meet legal requirements of their jurisdiction. Rescues and owners should be informed that documentation does not always guarantee imported dogs have adequately responded to vaccination and to seek veterinary advice.

**Summary**

- Nearly half of the imported dogs tested had inadequate rabies antibody titres (< 0.5 IU/mL) within a month of arrival in Canada, posing a risk to animal and public health. Of those with inadequate titres, 60% had no measurable rabies antibodies, suggesting they may have never been effectively vaccinated.
- Veterinarians should be aware of this risk and strongly consider revaccination of imported dogs to ensure they are adequately protected from rabies and comply with legal requirements in their jurisdiction.
- In contrast, the majority of the nonimported dogs tested for rabies antibodies had titres above the 0.5 IU/mL standard for international travel.

## 1 Introduction

Globally, rabies continues to pose a serious threat to animal and public health. Most human deaths from rabies are from exposure to rabid dogs, particularly in Asia and Africa, where canine‐variant rabies remains endemic (WHO 2024). Due to the prolonged incubation period for rabies, dogs infected with canine‐variant rabies have been unknowingly brought into areas free of canine rabies, including the United States (U.S.), European Union and Canada (Di Salvo et al. 2023; Hercules et al. 2018; Johnson et al. 2011; Lemmermann et al. 2025; Raybern et al. 2020; Rebellato et al. 2022; Whitehill et al. 2022). Canada reported two separate cases of canine‐variant rabies in dogs recently imported from Iran in 2021 and 2022 (Di Salvo et al. 2023; Rebellato et al. 2022).

Most dogs imported into Canada must have a current rabies vaccination certificate (Government of Canada 2024). This requirement does not eliminate the risk of importing a dog incubating canine rabies virus (Di Salvo et al. 2023) as dogs may be exposed prior to vaccination in the country of origin. Nevertheless, vaccination of dogs is a key component of preventing human cases of rabies (WHO 2024). Given the potential exposure from other rabies variants endemic in wildlife, rabies vaccination of all dogs in Canada is strongly encouraged, and is a legal requirement in the province of Ontario (Government of Canada 2023; Government of Ontario 2024). At the time of this study, Ontario regulations required dogs to be vaccinated with a rabies vaccine approved for use in Canada and administered by a veterinarian licensed to practice in Ontario (Government of Ontario 2023).

In addition to rabies vaccination, some countries, excluding Canada, require rabies antibody titre testing to verify that a dog has mounted an appropriate vaccine response prior to importation (Aubert 1992; WOAH 2024). The World Organisation for Animal Health (WOAH) has set an acceptable rabies antibody titre level of 0.5 IU/mL for the international movement of dogs (WOAH 2024). It is important to note that there is no proven protective titre level against rabies virus in any species, as protection involves other immunological factors besides antibodies that are not easily measured (Aubert 1992; Moore 2021). However, vaccination and pre‐existing antibodies greatly increase a dog's probability of surviving should they be exposed to rabies virus (Dodds et al. 2020; Moore 2021). There are several rabies antibody titre tests commercially available, but the only ones approved by WOAH for the importation of dogs are virus neutralisation (VN) tests, such as the fluorescent antibody virus neutralisation test (FAVN) and the rapid fluorescent foci inhibition test (RFFIT). These are functional tests that measure the ability of the rabies antibodies present to inactivate rabies virus (Ciconello et al. 2022). FAVN and RFFIT tests have been shown to produce comparable results (Briggs et al. 1998; Cliquet et al. 1998). Countries' import regulations often require these tests to be conducted at an approved or designated laboratory (CDC 2024; Government of Japan 2021). Other tests such as enzyme‐linked immunosorbent assays (ELISAs) are available, but are not approved for fulfilling the requirements for international movement of dogs (WOAH 2024). Due to differences in how they measure rabies antibodies, VN and ELISAs are not directly comparable (Ciconello et al. 2022).

Several factors have been shown to influence a dog's rabies antibody titre after vaccination. These factors include age, breed, vaccine product, number of prior rabies vaccinations, as well as time between vaccination and sampling (Berndtsson et al. 2011; Kennedy et al. 2007; Mansfield et al. 2004; Wallace et al. 2017; Zanoni et al. 2010). Despite these factors, most dogs are expected to produce an adequate titre (≥ 0.5 IU/mL) within a month of receiving a single rabies vaccine (Mansfield et al. 2004; Wallace et al. 2017; Yang et al. 2023). However, studies in multiple countries have reported 13%–73% of imported dogs did not have adequate titres when tested upon arrival despite arriving with rabies vaccination certificates (Kaila et al. 2019; Klevar et al. 2015; Nodari et al. 2017; Wright et al. 2023). Even more concerning, several dogs in these studies with inadequate titres had no measurable antibody response (< 0.1 IU/mL), which suggests they were never effectively vaccinated for rabies (Kaila et al. 2019; Klevar et al. 2015; Wright et al. 2023). The World Small Animal Veterinary Association acknowledges that vaccine husbandry remains an issue in many countries, and improper storage, handling, and administration (e.g., not storing at optimal temperature, exposure to extreme temperatures, using expired products, or not following label instructions) can diminish vaccine efficacy (Day et al. 2016; Ellis et al. 2024; PHAC 2007). In the U.S., serological testing of a group of 26 imported dogs revealed that 18 appeared to have not been recently vaccinated, contradicting their accompanying rabies vaccination certificates (Raybern et al. 2020).

There is little information on the rabies titres of dogs entering Canada because this testing is not an import requirement. The objective of this project was to determine the proportion of dogs with rabies antibody titres below the recommended 0.5 IU/mL in a sample of dogs imported with rabies vaccination certificates, and to compare this to rabies antibody titres in a population of dogs in Ontario, Canada, for which rabies antibody testing was performed independently of this study. An understanding of rabies antibody titres in imported dogs offers an opportunity to protect the health of imported dogs and the health of humans and animals with which they may have contact after their arrival.

## 2 Materials & Methods

This study was approved by the University of Guelph's Animals Care Committee (Animal Use Protocol #4717).

### 2.1 Recruitment and Sampling of Imported Dogs

Between October 2021 and November 2022, all dogs imported into Ontario, Canada, via either route (personal or commercial), from any country that were apparently healthy and could be sampled within 30 days of arrival were eligible to participate in the study. A convenience sample of imported dogs was recruited by contacting Ontario rescue organisations that were actively importing dogs from abroad into Ontario. These rescues were identified by either web search or were personal contacts of the authors and were contacted via email or phone. Rescues interested in participating chose to either have their newly imported dogs sampled by the research team upon the dog's arrival to Toronto's Pearson International Airport or to include a brochure about the study in each imported dog's information package provided to their new owners. If owners were interested in participating, the researchers contacted their veterinary clinic to coordinate sample collection and shipment.

At least 2 mL of serum was collected from each dog within 1 month of arrival and before they were revaccinated with a rabies vaccine in Canada. If dogs arrived in groups, no more than 10 dogs per group were sampled to limit clustering. Due to challenges in recruitment, this was adjusted to 15 dogs per group during the study. Additional serum samples stored from a previous project involving imported dogs were also included if the dog and sample collection met the inclusion criteria (Ontario Animal Health Network 2019).

Dog demographic and health data were collected with each sample, when available. This included breed, sex, age, reproductive status (intact or not), country of origin, date of arrival, date of sample collection, date of last rabies vaccination, and vaccine product administered. If rabies titre testing was completed abroad by the rescue organisation prior to importation, those data were also collected whenever possible.

Samples were stored at −80°C until all sampling was complete; then samples were shipped to the Rabies Laboratory of Kansas State Veterinary Diagnostic Laboratory (Kansas, United States) for RFFIT.

### 2.2 Rabies Titres From Nonimported Dogs

Records from the University of Guelph's Animal Health Laboratory were searched for rabies antibody titre tests from dogs submitted between 2013 and 2023. Information collected from these records included the type of rabies antibody titre test conducted (RFFIT or FAVN), rabies antibody titre results, sex, age, breed, reproductive status, client province and history provided by the submitting clinic. Dogs that had no accompanying history, or the history indicated that the dog had been imported, were excluded. Available histories were reviewed and the reason for titre testing was categorised as either pre‐export testing or assessment of the rabies antibody titre due to factors such as underlying disease (e.g., cancer, immune‐mediated disease), a previous reaction to vaccination, owner vaccine hesitancy, or discontinuation of the vaccine without further explanation. If provided, the date of the most recent rabies vaccine was also collected. No information on vaccine type or number of previous vaccines was available for this group.

### 2.3 Statistical Analysis

The signalment, history and rabies titre results from imported dogs and nonimported dogs were recorded in Microsoft Excel (Version 2302). For both datasets (imported and nonimported), frequencies were reported for categorical variables and medians and ranges were reported for continuous variables. For the nonimported dog dataset, FAVN and RFFIT titre value results were reported separately since they had different maximum detectable values reported (WOAH 2024). Rabies titre test results from RFFIT, FAVN, and ELISA tests were expressed as a binary variable based on whether or not the rabies titre value met the 0.5 IU/mL threshold. For both datasets, the interval from vaccination to sampling was determined in days. Rabies vaccines were categorised into those approved and not approved for use in dogs in Canada (Canadian Food Inspection Agency 2024; Government of Ontario 2024). For dogs with ELISA titres, the interval between ELISA titre testing and rabies vaccination was calculated in days. For dogs that had both ELISA and RFFIT titre testing, the number of days between sampling for these tests was determined. A Mann–Whitney U test was used to evaluate significant differences in the ages of the imported and nonimported dog groups.

For further analysis, certain variables were categorised. Age was categorised into less than 1 year and 1 year or greater. Breed was divided into purebred and mixed breed dogs. Purebred status was assigned when the dog's records indicated a single breed and mixed breed status was assigned when records indicated “mixed breed” or listed multiple breeds. The country of origin variable was categorised into regions of Asia, Africa and the Caribbean. The vaccination‐to‐sampling interval was categorised into less than or equal to 28 days and greater than 28 days.

Univariable logistic regressions were conducted using the imported dog dataset to explore age, breed, sex, and country of origin as predictors of whether dogs reached a rabies titre of at least 0.5 IU/mL (Y/N). Importer was not examined because it was similar to the country of origin variable and could not be further categorised for logistic analysis. For the subset of imported dogs with vaccine information, vaccine approval status and vaccine to sampling interval were also explored with the outcome of meeting the rabies titre of at least 0.5 IU/mL. Significance was set at α = 0.05. Univariable analysis was not performed on the nonimported dataset as there were fewer than 10 dogs with rabies antibody titres below 0.5 IU/mL. Data cleaning, visualisation, and analysis were done in R Statistical Software (Version 4.3.1; R Core Team, Vienna, Austria, 2023; https://www.R‐project.org) with RStudio (Version 2023.06.1.524; Posit Software, PBC, Boston, USA, 2023).

## 3 Results

### 3.1 Rabies Titres in Imported Dogs

**FIGURE 1:**
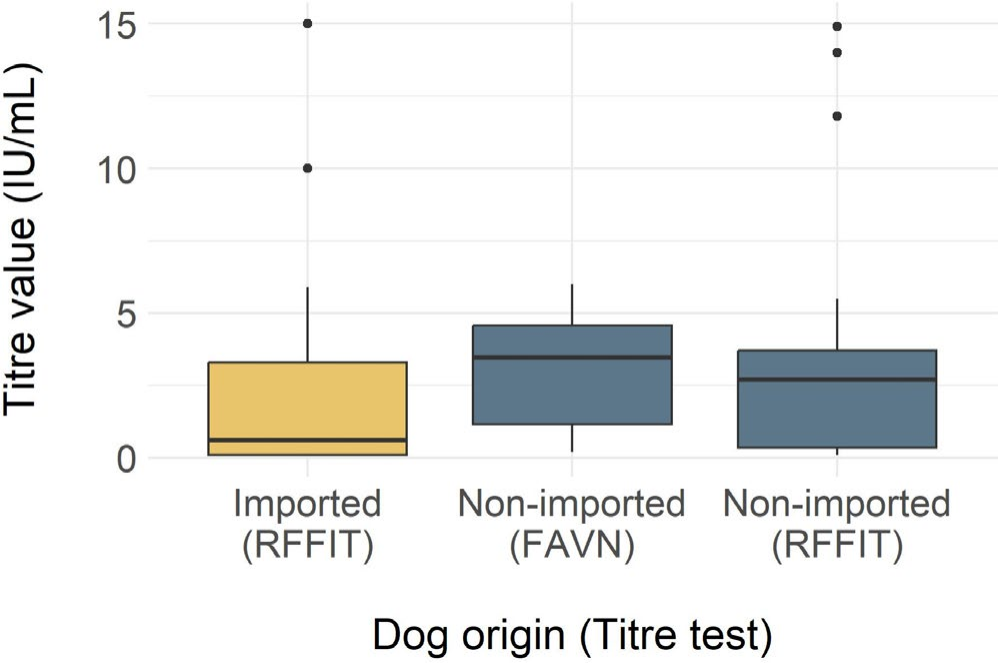
Distribution of rabies antibody titres from the 67 imported and 65 nonimported dogs. Rapid fluorescent foci inhibition test, RFFIT; fluorescent antibody virus neutralisation, FAVN.

**FIGURE 2:**
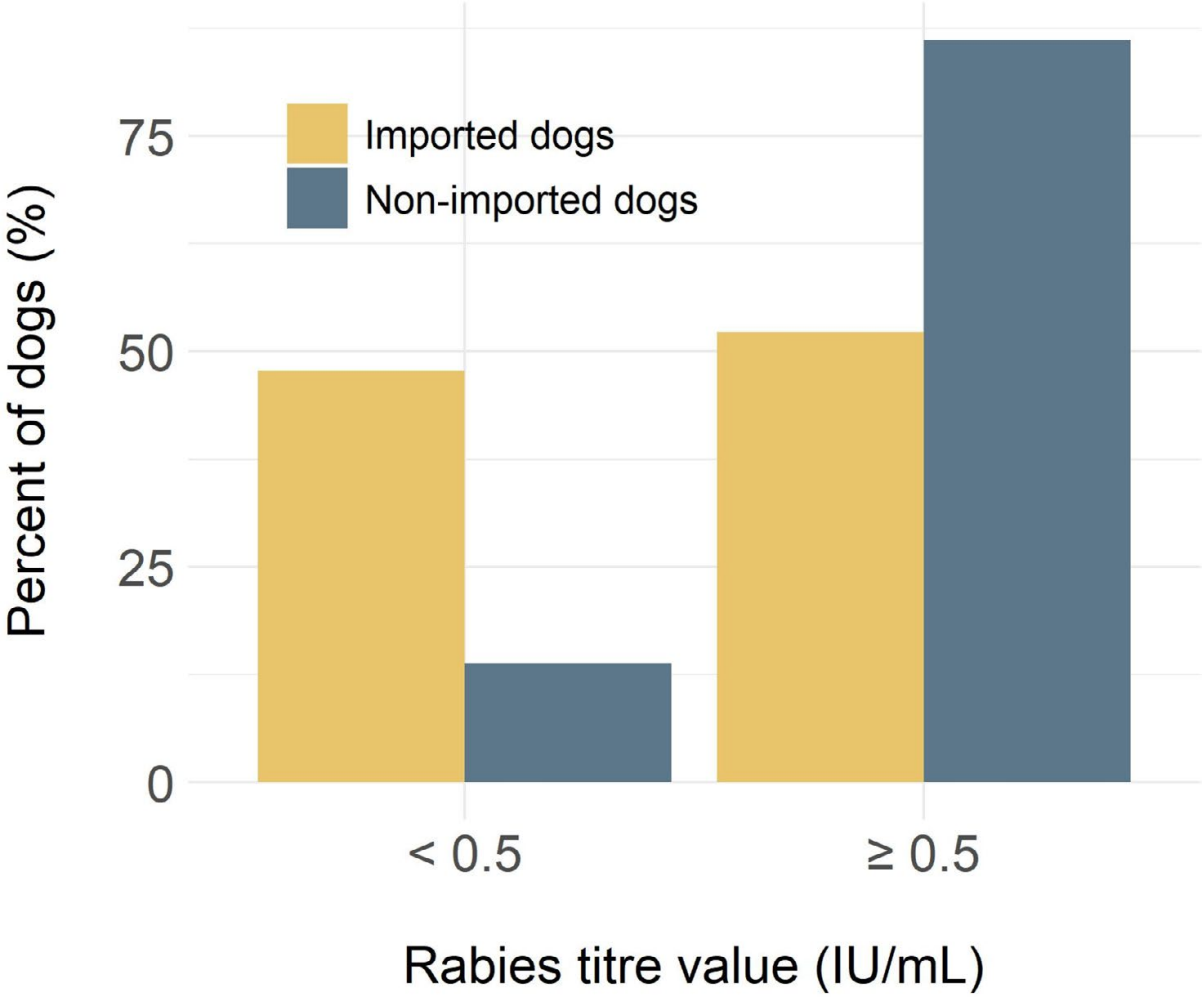
Percent of imported (*n* = 67) and nonimported (*n* = 65) dogs that had rabies antibody titres below 0.5 IU/mL or greater than or equal to 0.5 IU/mL.

In total, 67 imported dogs were included in the study, including 56 dogs sampled specifically for this study and 11 dogs that met the inclusion criteria that had banked serum samples. Imported dogs were mostly purebred (50/67, 75%), with a median age of 14 months, and included slightly more females (35/67, 52%) than males (29/67, 43%) and dogs of unknown sex (3/67, 4%) (Table 1). The dogs were imported by five rescue organisations and one humane society and originated from Egypt (46/67, 69%), China (9/67, 13%), the Bahamas (6/67, 9%), Antigua and Barbuda (2/67, 3%) and one (1/67, 2%) each from Azerbaijan, India, South Korea, and Turkey. The sampled dogs arrived as part of 17 separate importation events. The number of dogs sampled per event ranged from 1 to 15, with a median of three dogs. The largest samples, consisting of 12, 13 and 15 dogs, showed variability in rabies antibody titre results. Overall, the median rabies antibody titre (RFFIT) of the imported dog group was 0.6 IU/mL, with a range from less than 0.1 IU/mL to greater than 15 IU/mL (Figure 1). Almost half the imported dogs (32/67, 48%) did not meet the 0.5 IU/mL rabies titre threshold, and 19 (19/32, 60%) of the dogs that did not meet this threshold had no measurable titre (< 0.1 IU/mL) (Figure 2).

**TABLE 1:** Demographic variables of 67 imported and 65 non‐imported dogs for which rabies antibody titres were tested.

Demographic variable		Imported dogs *n* ^a^ (%)	Nonimported dogs *n* ^a^ (%)
Age	Median	14 months	5 years
	Range	3 months–8 years	4 months–14 years
Breed	Purebred	50 (75%)	40 (62%)
	Mixed	17 (25%)	15 (23%)
	Not reported	0	10 (15%)
Sex	Female	35 (52%)	25 (38%)
	Male	29 (43%)	33 (51%)
	Not reported	3 (4%)	7 (11%)
Reproductive status	Spayed	23 (34%)	10 (15%)
	Neutered	16 (24%)	11 (17%)
	Intact	8 (12%)	37 (57%)
	Not reported	20 (30%)	7 (11%)

Abbreviation: *n* ^a^, number of dogs within each category.

Rabies vaccination certificates were acquired from 26 of the 67 imported dogs (26/67, 39%). The median age for this group was 1 year (range 3 months to 8 years). The median number of days between rabies vaccination and sampling for the RFFIT titre was 42 days, with a range from 3 to 303 days. Four different rabies vaccines were listed on the certificates. Only one was approved for use in Canada, which was used in 46% of these dogs (12/26). The median titre for this subgroup was 0.3 IU/mL (range < 0.1–> 15 IU/mL). Half of these dogs (13/26, 50%) did not have a titre that met the 0.5 IU/mL threshold, and included seven that had titres below 0.1 IU/mL.

Results of rabies titre testing by ELISA completed prior to import were available for 29 dogs (29/67, 43%), all from Egypt. Testing was completed between 30 and 40 days after vaccination, with a median of 32.5 days. All 29 dogs had ELISA titres above 0.5 IU/mL, and the median titre was 1.2 IU/mL (range 0.5 IU/mL to 3.0 IU/mL). RFFIT testing was completed between 5 and 21 days following the ELISA, with a median of 7 days. When comparing RFFIT and ELISA titres in the same dogs, 11 dogs (11/29, 38%) had an ELISA titre above the 0.5 IU/mL threshold but an RFFIT below the 0.5 IU/mL threshold.

### 3.2 Rabies Titres in Nonimported Dogs

**FIGURE 3:**
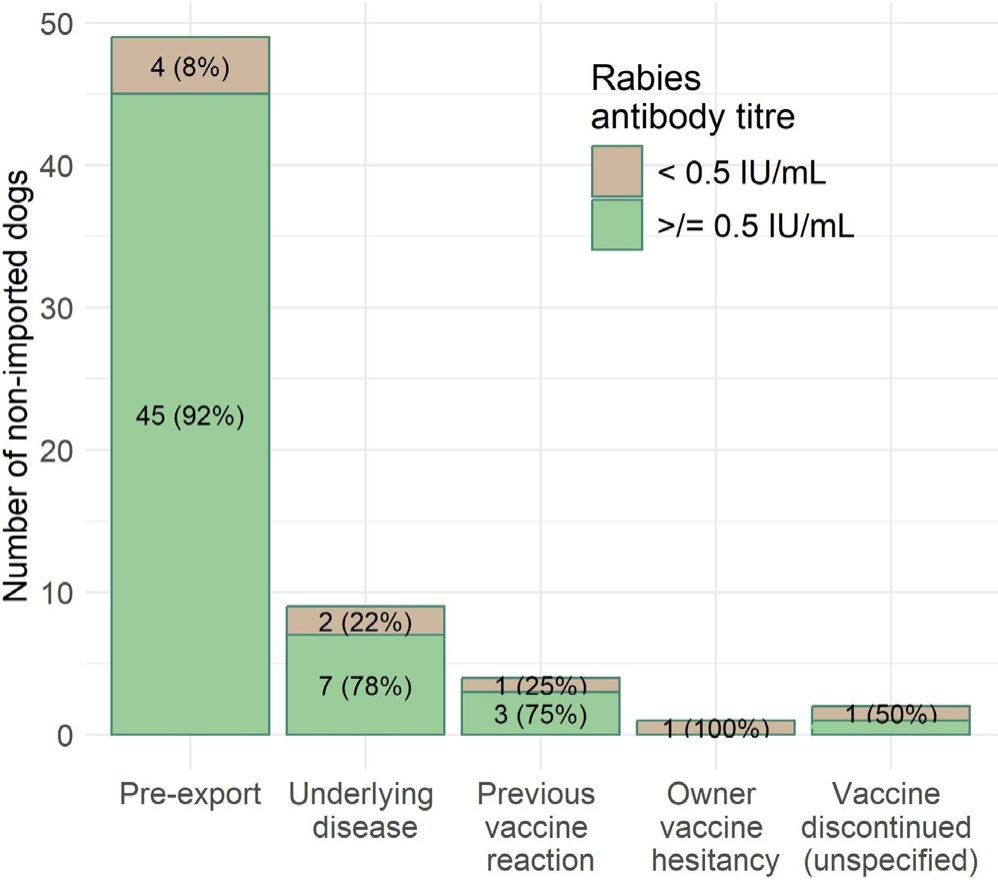
Reasons provided on laboratory submission for conducting rabies antibody titre testing in nonimported dogs at the Animal Health Laboratory (Guelph Ontario, Canada), categorised by test result (below 0.5 IU/mL or at or above 0.5 IU/mL).

Rabies antibody titres were available for 329 dogs tested through the Animal Health Laboratory. Records of 264 dogs were excluded due to lack of history (*n* = 197) or history of importation (*n* = 67). The records of the remaining 65 dogs were used for analysis. Nonimported dogs were mostly purebred (40/65, 62%), with a median age of 5 years, and included more males (33/65, 51%) than females (25/65, 38%) or dogs of unknown sex (7/65, 11%) (Table 1). All dogs were patients of clinics in Ontario (63/65, 97%), except for one (1/65, 1.5%) dog that was a patient of a clinic in Manitoba and one dog (1/65, 1.5%) that was a patient of a clinic in Quebec. Records indicated various rationales for titre testing (Figure 3). Most of the dogs had rabies titres completed using the FAVN (49/65, 75%) rather than the RFFIT (16/65, 25%). The median rabies titre of the nonimported group with the FAVN test was 3.46 IU/mL (range less than 0.2 IU/mL to greater or equal to 6.01 IU/mL) and the median rabies titre with the RFFIT was 2.70 IU/mL (range < 0.1–14.9 IU/mL) (Figure 1). Of the 65 dogs, 56 (86%) had titres at or above 0.5 IU/mL and 9 (14%) had titres below (Figure 2). Most dogs tested for pre‐export had rabies titres at or above 0.5 IU/mL (45/49, 92%). All other reasons for testing had larger proportions of dogs with inadequate rabies antibody titres (Figure 3). Three dogs had rabies antibody titres below the limit of detection and were tested due to previous vaccine reaction or as a pre‐export requirement. The date of the last rabies vaccine was available for nine dogs, with a median of 32 days between last vaccination and submission date (range 23–1374 days). All nine dogs had adequate rabies antibody titres. Seven of these dogs had their titre done as part of pre‐export testing and had a median of 31 days since their last rabies vaccination. The remaining two dogs had an underlying disease, and it had been over 3 years since their last rabies vaccine.

### 3.3 Statistical Analysis

No statistically significant associations were found for any variable and the outcome of a rabies antibody titre greater than or equal to 0.5 IU/mL (*p* > 0.05) (Table 2). There was a significant difference in the median age of dogs in the imported and nonimported groups (U = 893, *p* < 0.01), with imported dogs being younger than the nonimported group. Further comparisons between the two groups were not done due to limited information on the origin and status of the nonimported dogs.

**TABLE 2:** Results of univariable logistic regression analyses for associations with a rabies antibody titre of at least 0.5 IU/mL in 67 imported dogs.

Predictor variable		OR (95% CI)	*p*
Age (years)	≥ 1 year	0.63 (0.18–2.00)	0.44
	< 1 year^a^	Referent	
Sex	Male	0.7 (0.26–1.88)	0.48
	Female	Referent	
Breed	Purebred	2.53 (0.83–8.39)	0.11
	Mixed breed^a^	Referent	
Country of origin	Africa	2.27 (0.66–8.56)	0.20
	Caribbean^a^	0.96 (0.14–5.92)	0.97
	Asia^a^	Referent	
Vaccine status in Canada	Approved^a^	1.87 (0.40, 9.38)	0.43
	Not approved^a^	Referent	
Vaccine to sampling interval	≤ 28 days^a^	1 (0.15, 6.60)	1
	> 28 days	Referent	

^a^ Less than 10 observations in some cells.

## 4 Discussion

In this study, almost half of the imported dogs sampled within a month of their arrival to Canada had rabies antibody titres below 0.5 IU/mL, of which 60% had no measurable antibodies. This represents a higher‐than‐expected proportion of dogs with inadequate rabies titres after vaccination (Kennedy et al. 2007; Wallace et al. 2017; Zanoni et al. 2010). The fact that many imported dogs with inadequate titres had no measurable antibodies (< 0.1 IU/mL) suggests they may have never been effectively vaccinated, despite arriving with current rabies vaccination certificates. While a proven protective titre for dogs has not been established, many of these dogs may be insufficiently protected against rabies if exposed while in Canada. Immunity after rabies vaccination is critical for maintaining public and animal health (WHO 2024).

While various factors can influence a dog's response to vaccination, the majority of dogs are expected to develop an antibody response after a single rabies vaccine, which peaks at 15–30 days and gradually declines over the following months or year (Kennedy et al. 2007; Wallace et al. 2017; Zanoni et al. 2010). Explanatory factors including age, breed, vaccine approved, country of origin, and the interval between vaccination and sampling were investigated but showed no significant associations with a titre > 0.5 IU/mL. The study may have lacked sufficient power to detect these associations, or these results may have been driven by other unmeasured factors such as poor vaccine storage, poor vaccine quality, or improper administration of vaccines (Day et al. 2016; Ellis et al. 2024; PHAC 2007). Additionally, falsification of vaccine certificates in imported dogs has been confirmed or highly suspected in several shipments of dogs imported into the U.S. (E. Pieracci et al. 2022; E. G. Pieracci et al. 2021; Raybern et al. 2020) and was one of the reasons the U.S. implemented new regulations in 2024 requiring a serology test for dogs being imported from countries considered high‐risk for canine‐variant rabies (Bickell and Sekar 2025). Introduction of this requirement for dog importation in Canada could be one method of improving the vaccination status of imported dogs; however, it could also lead to greater costs, longer waiting periods, and potentially falsification of laboratory test results (Smith et al. 2021). Variability between laboratories and between rabies antibody titre test methods is also a potential challenge, as was evident in this study between the preimport ELISA and the subsequent RFFIT titres.

In contrast, most nonimported dogs (86%) had adequate rabies antibody titres. Dogs with inadequate titres in this group were most commonly those tested for reasons suggesting they had not been recently vaccinated. The lowest proportion of inadequate titres was in dogs tested for pre‐export. This is not surprising, as these dogs are likely to have been vaccinated more frequently and more recently. Additionally, the nonimported group was significantly older than the imported dog group, which may have also contributed to higher titres, as older dogs are more likely to have received multiple rabies vaccinations over their lifetime (Zanoni et al. 2010).

The findings of this study are not unique to Canada, with significant portions of imported dogs lacking adequate rabies antibody titres described in Finland, Sweden, Italy, the United Kingdom and U.S. (Kaila et al. 2019; Klevar et al. 2015; Nodari et al. 2017; Raybern et al. 2020; Wright et al. 2023). Most imported dogs in these studies originated from Eastern Europe, particularly Romania, whereas this study had a substantial number of dogs from Egypt. Also similar to this study, several of these previous studies reported much higher rabies antibody titres in local dog populations (Kaila et al. 2019; Klevar et al. 2015; Nodari et al. 2017).

As veterinarians are a critical point of contact with newly imported dogs (Belanger et al. 2025), they should be aware of the potential for low rabies antibody titres in imported dogs and the potential implications for lack of protection against rabies in these animals. To comply with Ontario rabies immunisation legislation, all the imported dogs in this study would have to be revaccinated, regardless of titre results (Government of Ontario 2024). This requirement does not exist in all provinces and highlights a need to educate veterinarians and importers about the value of re‐vaccination in jurisdictions where rabies vaccination of dogs is voluntary. Owners and rescue organisations should also be made aware of this gap and encouraged to have newly imported dogs seen by a veterinarian shortly after arrival for review of their medical records and to update vaccines and provide other preventative care and treatment, as appropriate (Belanger et al. 2025).

There are several limitations to this study. Recruitment of imported dogs was difficult as most rescue organisations declined or did not respond to inquiries to participate. Convenience sampling was heavily relied upon and may have introduced volunteer bias from the few rescues willing to participate. Moreover, sample sizes were small with limited diversity in the origin of dogs. This led to a sample population that is unlikely to be representative of all dogs being imported into Ontario, and a potential lack of independence of observations with dogs sampled in larger groups. It also reduced the ability to detect associations between explanatory variables and the outcome of a titre of at least 0.5 IU/mL. Further, accurate historical information was missing for many dogs. Vaccine information was only available for a subset of these dogs. The ages of imported dogs were likely estimates and there was no formal testing done to verify breed in any dog, thus potentially introducing misclassification bias of the explanatory variables. In‐depth examination of the pre‐import ELISA titres was not possible as this test is not comparable to the RFFFT or FAVN based on the test methodology. While the nonimported group was considered native to Canada, based on the information available, it cannot be assumed that these dogs represent the general dog population in Ontario. Based on their history, many of the dogs in this group likely received numerous rabies vaccines, regular health checks, and titre testing to ensure they remain eligible for travel or were maintaining sufficient rabies titres in the face of medical contraindications to revaccination. As such, comparative analyses were not done with the imported dog group. As is common with laboratory data, limited history was available for these dogs, including the date of their last rabies vaccination.

## 5 Conclusion

In this study, many dogs imported into Ontario, Canada had a low or unmeasurable amount of circulating rabies antibodies. This represents a significant animal and human health risk and highlights a potential lack of compliance with appropriate and effective vaccination of dogs prior to importation, despite regulatory requirements. In contrast, very few of the nonimported dogs had inadequate titres and occurred more often in dogs who were likely not vaccinated very recently. Veterinarians should be aware of these potential gaps and discuss the risks with rescue organisations and new pet owners. Veterinarians should also revaccinate dogs to comply with jurisdictional regulations, where applicable. Rescues and pet owners should be encouraged to present newly imported dogs to a veterinarian for review of the vaccination history and must be aware that international records that cannot be verified may not be reliable.

Further research examining rabies titres in imported dogs should focus on obtaining a more representative sample of imported dogs. Expanding testing to include dogs entering Ontario from the U.S. would be particularly relevant, as these dogs are not required to be revaccinated upon arrival, and a large proportion of imported dogs come to Canada from the U.S. (Blackmore et al. 2023). Further sampling of a more representative population of dogs vaccinated in Ontario would also be useful to aid in contextualising rabies titres in nonimported versus imported dogs.

## Conflicts of Interest

The authors declare no conflicts of interest.

## Data Availability

The data that support the findings of this study are openly available in Borealis at https://doi.org/10.5683/SP3/6HUVRH.
